# Analysis of Hip Fractures in France During the First COVID-19 Lockdown in Spring 2020

**DOI:** 10.1001/jamanetworkopen.2021.34972

**Published:** 2021-11-17

**Authors:** Julien Paccou, Xavier Lenne, Grégoire Ficheur, Didier Theis, Bernard Cortet, Amélie Bruandet

**Affiliations:** 1 Department of Rheumatology, Centre Hospitalier Universitaire de Lille, University Lille, Lille, France; 2 Department of Medical Information, Lille University Hospital, Lille, France; 3 Évaluation des Technologies de Santé et des Pratiques Médicales, Centre Hospitalier Universitaire de Lille, University Lille, Lille, France

## Abstract

**Importance:**

The COVID-19 pandemic has posed a number of unprecedented challenges to the health care system in France, where hip fractures in the elderly population are a major public health concern.

**Objective:**

To explore the association of the first nationwide COVID-19 lockdown in France with the absolute number of hip fractures among patients 50 years or older.

**Design, Setting, and Participants:**

This retrospective cohort study used data from the French national hospitals database to identify patients 50 years or older who were hospitalized for hip fracture in France from January to July 2019 and January to July 2020.

**Exposures:**

The first nationwide COVID-19 lockdown in France from March 16 to May 10, 2020.

**Main Outcomes and Measures:**

The main outcome was the number of hospitalizations for hip fracture from January to July 2020 (study period) compared with the number of hospitalizations for hip fracture during the same period in 2019 (control period). Hospitalization rate ratios (HRRs) comparing the study period with the control period were calculated for 3 intervals (before lockdown [January 1 to March 15], during lockdown [March 16 to May 10], and after lockdown [May 11 to July 31]) and were stratified by gender, age and hospital type.

**Results:**

The study included 46 393 patients hospitalized for hip fracture during January to July 2019 (34 589 [74.4%] women; mean [SD] age, 82.8 [10.5] years) and 44 767 patients hospitalized for hip fracture from January to July 2020 (33 160 [74.1%] women; mean [SD] age, 82.9 [10.5] years). During the lockdown in 2020, 10 429 patients (23.30%) were hospitalized for hip fracture compared with 11 782 patients (25.40%) during the same period in 2019 (HRR, 0.89; 95% CI, 0.86-0.91; *P* < .001). The lockdown period was associated with a decrease in the number of hip fractures of 11% among women (from 8756 in 2019 to 7788 in 2020) and 13% among men (from 3026 in 2019 to 2641 in 2020). When the absolute number of hip fractures was stratified by age group, the lockdown period was associated with a decrease in the number of hip fractures in all age groups except in patients older than 89 years (HRR, 0.97; 95% CI, 0.92-1.01; *P* = .17). In the group of patients aged 80 to 89 years, the number of hip fractures decreased from 4925 to 4370 (HRR, 0.89; 95% CI, 0.85-0.92; *P* < .001). During the lockdown, hospitalizations decreased by 33% (HRR, 0.67; 95% CI, 0.63-0.71; *P* < .001) in public university hospitals and by 24% (HRR, 0.76; 95% CI, 0.73-0.79; *P* < .001) in public general hospitals but increased by 46% (HRR, 1.46; 95% CI,1.38-1.54; *P* < .001) in private for-profit hospitals.

**Conclusions and Relevance:**

In this cohort study, hospitalizations for hip fractures in France decreased by 11% during the first nationwide COVID-19 lockdown. Further studies are needed to investigate the long-lasting consequences of the COVID-19 pandemic on the incidence of osteoporotic fractures.

## Key Points

**Question:**

Was the first nationwide COVID-19 lockdown in France associated with a change in the absolute number of hip fractures among patients 50 years or older?

**Findings:**

In this cohort study of 46 393 and 44 767 patients 50 years or older with hip fracture in France from January to July 2019 and January to July 2020, respectively, the absolute number of hip fractures decreased by 11% during the first nationwide COVID-19 lockdown from March 16 to May 10, 2020, compared with the same period in 2019.

**Meaning:**

The findings suggest that the first nationwide COVID-19 lockdown in France was associated with a decrease in the absolute number of hip fractures.

## Teaser

This cohort study uses data from the French national hospitals database to compare the number of hip fractures among patients 50 years or older during the first nationwide COVID-19 lockdown in France in spring 2020 with the number during the same period in 2019.

## Introduction

The COVID-19 pandemic is a public health emergency of international concern. The most common symptoms of COVID-19 are fever, dry cough, and tiredness.^1,2^ Under guidance from the World Health Organization, many countries have implemented lockdowns to control the spread of the disease. France implemented its first strict lockdown from March 16 to May 10, 2020. During the lockdown, emergency legislation was introduced, confining people to their homes and restricting outdoor movement to essential activities, including medical care. In hospitals, to meet the increasing demands on the health care system owing to the influx of patients with COVID-19, only essential health care services were maintained.

The treatment of patients with osteoporosis during the first nationwide lockdown in France was challenging.^3^ Approximately 75 000 hip fractures are sustained in France each year.^4^ Hip fractures are the most common type of fragility fracture, and because they are associated with high morbidity and mortality rates, these injuries almost always require hospital admission and surgical management.^5,6^ It is not yet known whether the number of hip fractures was affected by the restrictions on outdoor movement imposed by the lockdown.^7^ As demonstrated in the multinational Global Longitudinal Study of Osteoporosis in Women,^8^ hip fractures in postmenopausal women occur equally inside and outside the home, and the nationwide lockdown in France may have been indirectly associated with either an increase or a decrease in the number of hip fractures. A few reports^9,10,11^ have suggested that either a decrease or no change in the number of patients presenting to hospitals with hip fractures occurred. However, discordant findings highlight the limitations of single-center studies involving a limited number of patients.

Considering these knowledge gaps, a larger population-based study including a large number of patients was needed. We conducted this nationwide cohort study with the hypothesis that the number of hip fracture–related hospitalizations among patients 50 years or older would decrease during the first nationwide COVID-19 lockdown in France in association with the changes in social behavior and mobility caused by the lockdown. Our main objective was to explore the association between the first nationwide COVID-19 lockdown and the number of hip fractures. Our secondary objectives were to perform stratified analyses by age group, gender, and hospital type (public or private).

## Methods

### Data Sources

This retrospective cohort study was conducted using data obtained from the Programme de Médicalisation des Systèmes d’Information (PMSI) database, which is the French national hospitals database. The PMSI is a comprehensive, all-inclusive database that is populated with information on all inpatient visits based on standardized hospital discharge reports in which all diagnoses and medical procedures are carefully recorded and summarized using a standardized coding system. In France, the coding and reporting of this information is mandatory for all hospital departments. Discharge report abstracts included patients’ demographic characteristics, diagnosis codes (based on the *International Statistical Classification of Diseases and Related Health Problems, Tenth Revision [ICD-10]*), and medical procedures (based on the Classification Commune des Actes Médicaux). The University of Lille institutional review board waived the need for approval and informed consent because the study used deidentified data and did not involve human participant research. This study followed the Strengthening the Reporting of Observational Studies in Epidemiology (STROBE) reporting guideline.

To ensure the quality of the data, tests are routinely performed when information on inpatient stays is sent to the French public health insurance agency. These tests include checks on the chronology of the hospital stays, the format (missing, incorrect, or inaccurate values) of the demographic characteristics (gender, age, date, mode of entry, and date and mode of discharge), the format and accuracy of procedure and diagnosis codes, and the consistency among procedure codes, diagnosis codes, length of hospital stay, age, and gender.

### Participants

The study included patients 50 years or older hospitalized for hip fracture in metropolitan France from January 1 to July 31, 2020 (study period), and from January 1 to July 31, 2019 (control period). Hip fractures were considered if they were identified with *ICD-10* primary diagnosis codes S7200 (unspecified femoral neck fracture), S7210 (unspecified trochanteric hip fracture), and S7220 (subtrochanteric fracture: closed).

**Figure 1.**
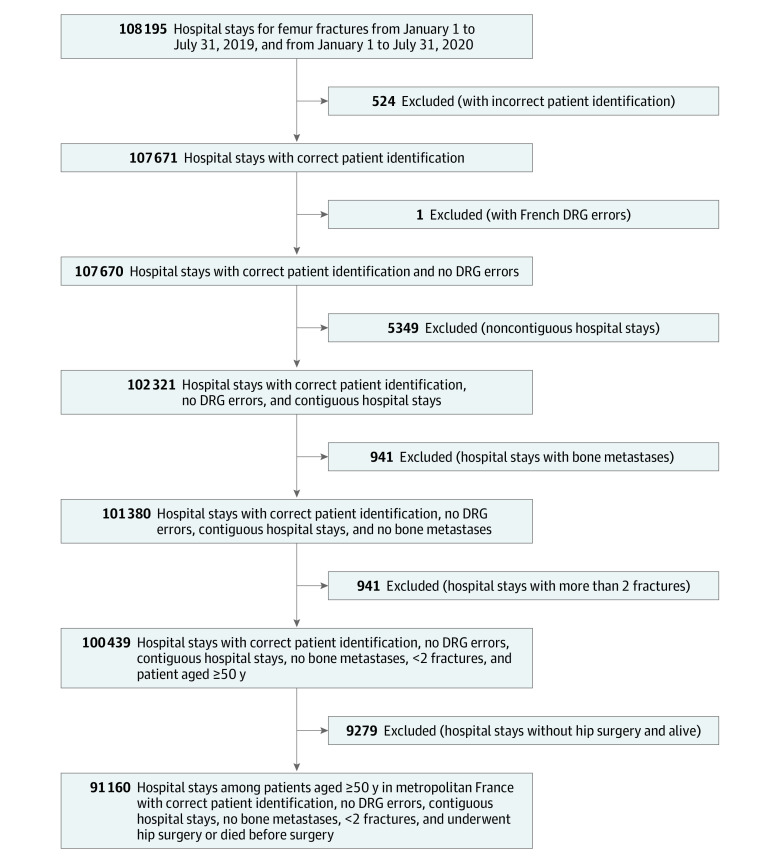
Study Flow Diagram Contiguous hospital stays indicate consecutive stays when the interval between stays was less than 1 day, and noncontiguous hospital stays indicate stays when the interval between stays was 1 day or more. DRG indicates diagnosis-related group.

The exclusion criteria were (1) age less than 50 years, (2) incorrect patient identifier, (3) incorrect French diagnosis-related group, (4) bone metastases during hospitalization for fracture, (5) presence of more than 2 fractures, and (6) no hip surgery (procedure codes are given in eTable 1 in the Supplement). To take into account the episode of care for the fracture, consecutive hospital stays for which the interval between stays was less than 1 day were considered as single care sequences. The starting date of the care sequence was the starting date of the first stay, and the end date was the date of death or the date of discharge at the end of the last stay (Figure 1).

### Exposures of Interest

The main exposure of interest was the year 2020 vs the year 2019. For the 2 years of the study, 3 intervals were considered: January 1 to March 15 (before the lockdown period), March 16 to May 10 (during the lockdown period), and May 11 to July 31 (after the lockdown period). Patients' gender, age, and hospital status were reported as well as whether the patient died during hospitalization.

### Main Outcome

The main outcome was the first hip fracture–related hospitalization in France from January 1 to July 31, 2020. Moreover, when PMSI data were used and the same inclusion and exclusion criteria as those used for the main outcome were applied, trends in the number of hip fractures sustained by women and men from 2015 to 2019 were estimated to evaluate changes.

For the lockdown period, all-cause mortality rate ratios (MRRs; hospital and nonhospital mortality) were compared with the hospitalization rate ratios (HRRs) for hip fracture. The number of deaths was obtained from the French National Institute of Statistics and Economic Studies, which regularly publishes the number of daily deaths (from all causes) in each French department.^12^ Other clinical factors, such as the Charlson Comorbidity Index, type 2 diabetes, chronic obstructive pulmonary disease, cardiovascular disease, and any osteoporotic fracture (excluding craniofacial, cervical spine, hand, finger, foot, and toe fractures), occurring within the 2 years before or during the hospital stay were identified using *ICD-10* codes (eTable 1 in the Supplement).

### Statistical Analysis

Categorical variables were expressed as numbers and percentages, and continuous variables were expressed as means and SDs. Hospitalization rate ratios comparing the study period with the control period were calculated for the 3 aforementioned intervals: before lockdown, during lockdown, and after lockdown. The secondary outcomes were the changes in hospitalization rates stratified by gender, age group (50-59, 60-69, 70-79, 80-89, and ≥90 years), and hospital type (public, public university, private for-profit, and private nonprofit). For each interval and each modality of secondary outcome variables, HRRs comparing the study period with the control period were calculated using Poisson regression to model the overall number of hospitalizations (eMethods in the Supplement).

To assess whether hospitalization rates for hip fracture during the lockdown period were associated with mortality (from all causes), we assessed overall HRRs and MRRs in each French department. Overall HRRs and MRRs comparing the study period with the control period were calculated using Poisson regression. Significance was considered at 2-tailed *P* < .05. All statistical analyses were performed using Stata, version 13 (StataCorp LLC).

## Results

### Trends in Hip Fractures in France From 2015 to 2019

Table 1 shows the annual number of hip fractures among women and men 50 years or older from 2015 to 2019. Overall, the number of hip fractures increased from 74 844 in 2015 to 79 340 in 2019. The 30-day hospital mortality rates among adults undergoing hip fracture surgery remained stable, ranging from 2.58% (1934 patients) in 2015 to 2.37% (1881 patients) in 2019.

**Table 1.** Annual Number of Hip Fractures and 30-Day Mortality in France From 2015 to 2019

Characteristic	Patients with hip fracture, No. (%)					Change between 2015 and 2019, No. (%)
	2015	2016	2017	2018	2019	
All	74 844 (100)	75 971 (100)	77 541 (100)	78 432 (100)	79 340 (100)	4496 (6.0)
Gender						
Men	18 697 (24.98)	18 918 (24.90)	19 661 (25.36)	19 910 (25.39)	20 235 (25.50)	1538 (8.2)
Women	56 147 (75.02)	57 053 (75.10)	57 880 (74.64)	58 522 (74.61)	59 105 (74.50)	2958 (5.3)
Age group, y						
50-59	2830 (3.78)	2848 (3.75)	2921 (3.77)	3029 (3.86)	2975 (3.75)	145 (5.1)
60-69	6324 (8.45)	6558 (8.63)	6825 (8.80)	6922 (8.83)	7054 (8.89)	730 (11.5)
70-79	11 759 (15.71)	11 892 (16.65)	12 534 (16.16)	13 007 (16.58)	13 320 (16.79)	1561 (13.3)
80-89	33 860 (45.24)	33 587 (44.21)	33 630 (43.37)	33 174 (42.30)	32 960 (41.54)	–900 (–2.7)
≥90	20 071 (26.82)	21 086 (27.76)	21 631 (27.90)	22 300 (28.43)	23 031 (29.03)	2960 (14.7)
Died within 30 d						
All^a^	1934 (2.58)	1933 (2.54)	1989 (2.57)	1893 (2.41)	1881 (2.37)	–53 (–2.7)
Gender^b^						
Men	782 (4.18)	772 (4.08)	826 (4.20)	752 (3.77)	756 (3.74)	–26 (–3.3)
Women	1152 (2.05)	1161 (2.03)	1163 (2.01)	1141 (1.95)	1125 (1.90)	–27 (–2.3)

^a^ The denominator was the total number of patients with hip fracture in the column.

^b^ The denominator was the total number of patients with hip fracture in each respective gender category in the column.

### Hip Fractures Before, During, and After the First Nationwide COVID-19 Lockdown in Spring 2020

In France, 44 767 hip fractures were recorded from January through July 31, 2020. A total of 33 160 hip fractures (74.07%) occurred among women, and the mean (SD) age of all patients with hip fracture was 82.9 (10.5) years. A total of 16 729 hip fractures occurred during the prelockdown period, 10 429 during the lockdown period, and 17 609 during the postlockdown period. Detailed descriptions of patients' characteristics before, during, and after the nationwide COVID-19 lockdown in the 2020 study period and during the 2019 control period are provided in Table 2.

**Table 2.** Patients' Characteristics Before, During, and After the First Nationwide COVID-19 Lockdown in France

Characteristic	Patients with hip fracture^a^					
	Before lockdown		During lockdown		After lockdown	
	2019	2020	2019	2020	2019	2020
All	17 023 (100)	16 729 (100)	11 782 (100)	10 429 (100)	17 588 (100)	17 609 (100)
Gender						
Men	4302 (25.27)	4390 (26.24)	3026 (25.68)	2641 (25.32)	4526 (25.73)	4576 (25.99)
Women	12 721 (74.73)	12 339 (73.76)	8756 (74.32)	7788 (74.68)	13 062 (74.27)	13 033 (74.01)
Age, mean (SD), y	82.8 (10.6)	82.8 (10.6)	82.9 (10.3)	83.7 (10.0)	82.7 (10.5)	82.7 (10.6)
Age group, y						
50-59	689 (4.05)	662 (3.96)	415 (3.52)	303 (2.91)	677 (3.85)	671 (3.81)
60-69	1511 (8.88)	1471 (8.79)	1024 (8.69)	794 (7.61)	1635 (9.30)	1634 (9.28)
70-79	2787 (16.37)	2807 (16.78)	1988 (16.87)	1645 (15.77)	2930 (16.66)	3008 (17.08)
80-89	7078 (41.58)	6879 (41.12)	4925 (41.80)	4370 (41.90)	7340 (41.73)	7240 (41.12)
≥90	4958 (29.13)	4910 (29.35)	3430 (29.11)	3317 (31.81)	5006 (28.46)	5056 (28.71)
Diabetes	799 (4.69)	805 (4.81)	563 (4.78)	461 (4.42)	895 (5.09)	828 (4.70)
COPD	2361 (13.87)	2391 (14.29)	1604 (13.61)	1440 (13.81)	2456 (13.96)	2372 (13.47)
CVD	942 (5.53)	923 (5.52)	683 (5.80)	605 (5.80)	986 (5.61)	942 (5.35)
Charlson Comorbidity Index						
0	7410 (43.53)	7439 (44.47)	5239 (44.47)	4474 (42.90)	7904 (44.94)	8040 (45.66)
1	4154 (24.40)	4058 (24.26)	2885 (24.49)	2586 (24.80)	4181 (23.77)	4238 (24.07)
2	2159 (12.68)	2088 (12.48)	1447 (12.28)	1303 (12.49)	2046 (11.63)	2106 (11.96)
≥3	3300 (19.39)	3144 (18.79)	2211 (18.77)	2066 (19.81)	3457 (19.66)	3225 (18.31)
History of any osteoporotic fracture before the index date	1700 (9.99)	1605 (9.59)	1111 (9.43)	917 (8.79)	1659 (9.43)	1415 (8.04)
Type of hospital						
Public university	3172 (18.63)	3127 (18.69)	2180 (18.50)	1457 (13.97)	3176 (18.06)	3087 (17.53)
Other public	9885 (58.07)	9693 (57.94)	6897 (58.54)	5243 (50.27)	10 478 (59.57)	9994 (56.76)
Private for-profit	3254 (19.12)	3166 (18.93)	2176 (18.47)	3171 (30.41)	3188 (18.13)	3682 (20.91)
Private nonprofit	712 (4.18)	743 (4.44)	529 (4.49)	558 (5.35)	746 (4.24)	846 (4.80)
Death during hospitalization	655 (3.85)	654 (3.91)	407 (3.45)	432 (4.14)	607 (3.45)	587 (3.33)
Type of fracture						
Femoral neck	10 527 (61.84)	10 048 (60.06)	7167 (60.83)	6260 (60.02)	10 672 (60.68)	11 028 (62.63)
Intertrochanteric	5692 (33.44)	5804 (34.69)	4063 (34.48)	3692 (35.40)	6015 (34.20)	5845 (33.19)
Subtrochanteric	804 (4.72)	877 (5.24)	552 (4.69)	477 (4.57)	901 (5.12)	736 (4.18)

Abbreviations: COPD, chronic obstructive pulmonary disease; CVD, cardiovascular disease.

^a^ Data are presented as number (percentages) of patients unless otherwise indicated.

### Comparison of Hip Fractures in France in 2020 vs 2019

**Figure 2.**
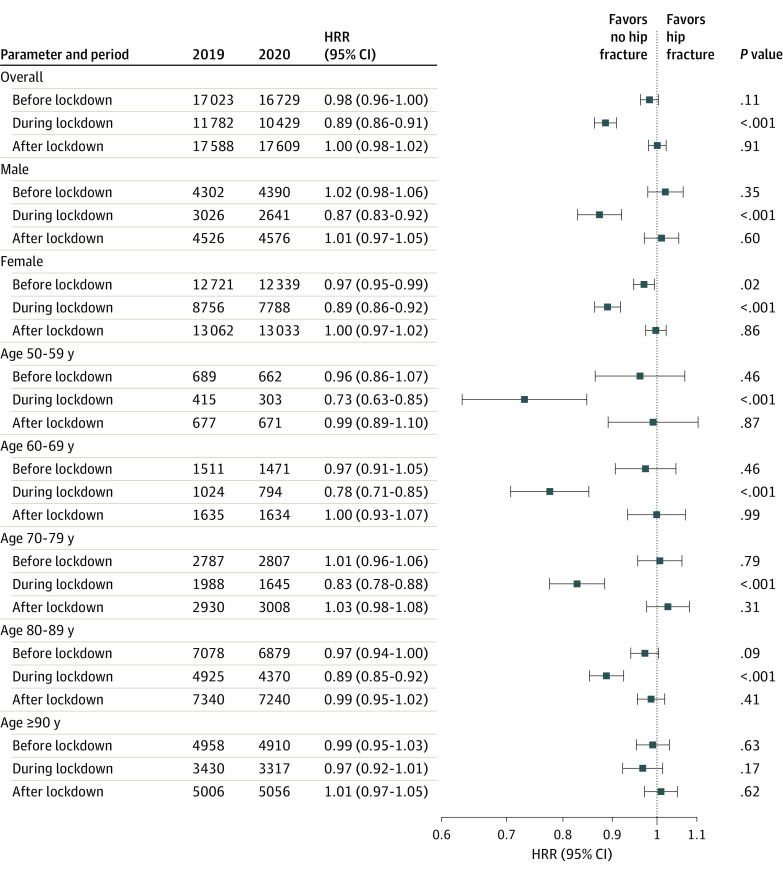
Hospitalization Rate Ratios (HRRs) of Hip Fractures by Age and Gender From January 1 to July 31, 2019, and From January 1 to July 31, 2020

The study included 46 393 patients hospitalized for hip fracture during January to July 2019 (34 539 [74.45%] women; mean [SD] age, 82.8 [10.5] years). During the nationwide COVID-19 lockdown period, 10 429 patients (7788 women [74.68%]; mean [SD] age, 83.7 [10.0] years) were hospitalized with hip fracture compared with 11 782 patients (8756 women [74.32%]; mean [SD] age, 82.9 [10.3] years) during the same period in 2019 (HRR, 0.89; 95% CI, 0.86-0.91; *P* < .001). Figure 2 shows the number of hip fractures in France from January 1 through July 31, 2020, compared with the same period in 2019 as well as the corresponding HRRs. Patients’ characteristics, comorbidities, and type of hip fracture did not differ between the 2 periods (Table 2). The number of hip fractures did not change significantly before or after the lockdown compared with the numbers in 2019; HRRs were 0.98 (95% CI, 0.96-1.00; *P* = .11) before the lockdown and 1.00 (95% CI, 0.98-1.02; *P* = .91) after the lockdown.

### Analyses Stratified by Gender and Age Group

During the lockdown period, hospitalizations decreased among both men and women; HRRs were 0.87 (95% CI, 0.83-0.92; *P* < .001) for men and 0.89 (95% CI, 0.86-0.92; *P* < .001) for women (Figure 2). The number of hospitalizations did not change significantly before or after the lockdown in 2020 compared with 2019 but decreased slightly among women during the prelockdown period (from 12 721 in 2019 to 12 339 in 2020; HRR, 0.97; 95% CI, 0.95-0.99; *P* = .02).

When the absolute number of hip fractures was stratified by age group, the lockdown period was associated with a decrease in the number of hip fractures in 2020 compared with 2019 among all age groups except patients older than 89 years (HRR, 0.97; 95% CI, 0.92-1.01; *P* = .17), among whom the number of hip fractures was 3317 in 2020 compared with 3430 in 2019. In the group of patients aged 80 to 89 years, who had the highest number of hip fractures, hip fractures decreased from 4925 in 2019 to 4370 in 2020 (HRR, 0.89; 95% CI, 0.85-0.92; *P* < .001). In the youngest age group (50-59 years), the number of hip fractures decreased from 415 in 2019 to 303 in 2020 (HRR, 0.73; 95% CI, 0.63-0.85; *P* < .001) (Figure 2). Overall, HRRs during the lockdown period increased as age increased.

### Analyses Stratified by Hospital Type 

**Figure 3.**
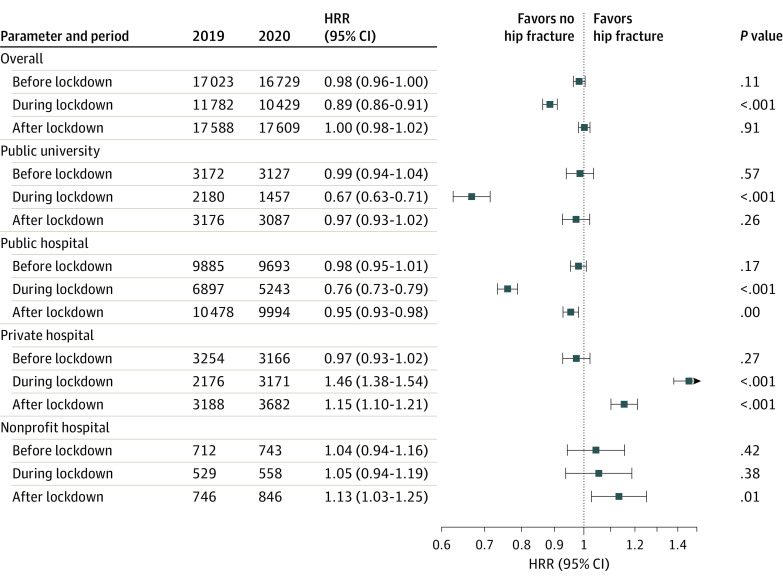
Hospitalization Rate Ratios (HRRs) of Hip Fractures by Hospital Type From January 1 to July 31, 2019, and From January 1 to July 31, 2020

Figure 3 shows the number of hip fractures according to hospital type from January 1 through July 31, 2020, compared with the same period in 2019 as well as the corresponding HRRs. Compared with 2019, during the lockdown, hospitalizations decreased by 33% (HRR, 0.67; 95% CI, 0.63-0.71; *P* < .001) in public university hospitals and by 24% (HRR, 0.76; 95% CI, 0.73-0.79; *P* < .001) in public general hospitals but increased by 46% (HRR, 1.46; 95% CI, 1.38-1.54; *P* < .001) in private for-profit hospitals. Compared with 2019, during the postlockdown period, hospitalizations decreased by 5% (HRR, 0.95; 95% CI, 0.93-0.98; *P* = .001) in public general hospitals but increased by 15% (HRR, 1.15; 95% CI, 1.10-1.21; *P* < .001) in private for-profit hospitals and by 13% (HRR, 1.13; 95% CI, 1.03-1.25; *P* = .01) in private nonprofit hospitals. Pearson analysis showed no correlation (*r* = –0.030; *P* = .78) between all-cause MRRs in the French departments and HRRs for hip fracture during the lockdown period (eFigure in the Supplement).

## Discussion

In this cohort study using data from the French national hospital database, the absolute number of hip fractures decreased by 11% during the first nationwide COVID-19 lockdown during March 16 to May10, 2020, compared with the same period in 2019. The lockdown period was associated with a decrease in the number of hip fractures among women (11%) and men (13%). When the absolute number of hip fractures was stratified by age group, the lockdown period was associated with a decrease in the number of hip fractures in all age groups except patients older than 89 years. During the lockdown period, HHRs decreased in public university hospitals and public hospitals but increased in private for-profit hospitals.

To our knowledge, this is the first study to provide detailed national data on hip fractures during the COVID-19 lockdown in France. Although authors in the UK^9,10^ observed a similar rate of hip fractures, studies from Spain,^11^ Poland,^13^ and Italy^14,15^ reported lower rates. In a cross-sectional study in Edinburgh,^9^ hip fracture–related hospital admissions remained unchanged during March 5 to April 13, 2019, and during the same period in 2020. The mean number of daily orthopedic referrals in 2019 was 3.0 (95% CI, 2.5-3.5) compared with 2.7 (95% CI, 2.2-3.1) in 2020 (*P* = .50). In an observational study in Nottingham,^10^ data on fracture clinic attendance during lockdown were compared with respective data from the same period used in this study (ie, 2015-2019). During lockdown, the number of hip fractures remained unchanged; a mean (SD) of 16.1 (5.6) inpatients per week were admitted for hip fracture, which was similar to the number of admissions in previous years.^10^ A study in Madrid^11^ evaluated the number and characteristics of patients with hip fractures admitted to hospitals during the COVID-19 lockdown. The patients' characteristics were similar to those in our study, but the number of hip fractures decreased by approximately one-fourth; 64 patients were admitted during the lockdown period in 2020, and 172 were admitted during the corresponding periods in 2018 (82 patients) and 2019 (90 patients). A study conducted in Poland^13^ assessed whether the COVID-19 pandemic was associated with the number of hip fractures. The number of hip fractures reported during the 77-day period of observation was 29, whereas the mean number of hip fractures reported for the corresponding periods from 2015 to 2019 was 33. The annual incidence rate of hip fracture decreased by 13.4% per 100 000 inhabitants compared with the previous years. In a study in Piacenza and Parma, Italy,^14^ the number of proximal femur fractures also decreased significantly by 28.4% during the COVID-19 pandemic from 169 fractures treated from February through March 2019, to 121 during the same period in 2020. In another study^15^ conducted in Italy during the COVID-19 pandemic that involved 15 orthopedic and trauma units (public and private hospitals throughout the country), the authors reported a stable reduction of 15% to 20% in the number of surgical interventions to treat femoral neck fractures compared with numbers during the same timeframe of the previous year (2019).

Local rates and the severity of lockdown measures as well as observation periods may have affected the reported findings in these studies. In the studies conducted in Edinburgh^9^ and Madrid,^11^ the observation periods were 40 and 61 days, respectively, and in the studies conducted in Northern Italy^14^ and Nottingham,^10^ the observation periods were 57 and 49 days, respectively. The reason that some authors observed a reduction in admissions for fragility hip fracture and others did not is unclear.^16^ To our knowledge, periods of lockdown similar to those enforced during the COVID-19 pandemic have never previously occurred. The changes in social behavior and mobility exhibited during the early stages of the COVID-19 pandemic may explain our findings.^7^ Our results correspond with the unexplained reductions in hospital admissions for common emergencies such as myocardial infarction and stroke.^17,18^ Some researchers^17,18^ have postulated that the reductions in admissions for myocardial infarction and stroke were associated with fear of in-person treatment during the COVID-19 pandemic, causing people to stay at home rather than to seek treatment. However, with regard to hip fractures, this hypothesis seems unlikely because surgical treatment cannot be delayed because of the major functional effects of hip fractures and the pain associated with them. Another possible explanation is that the lack of outdoor activity during the first nationwide lockdown was associated with lower fall rates. Because many falls occur outdoors during normal day-to-day activities, the stay-at-home strategy may have been associated with a lower number of hip fractures.

Our results revealed a differential association of age with the number of hip fractures during the lockdown period. A pattern was observed in which the HRRs during the lockdown period increased as age increased. Older participants may have been more resistant to lockdown policies because the outdoor-to-indoor hip fracture ratio may have been different according to age and gender.^8,13^ The Global Longitudinal Study of Osteoporosis in Women population^13^ consisted of ambulatory postmenopausal women (median age, 77 years) who may have been more health conscious and active than those in our study, and hip fractures reported in that study (n = 231) occurred equally inside and outside the home. Of note, we did not have enough data regarding the place of occurrence of hip fractures according to patients' gender and age.

We showed that the first nationwide COVID-19 lockdown in France had a differential association with the number of hip fractures depending on hospital type. The overburdening of the public health care system by patients with COVID-19 may explain these findings. Patients with hip fracture who are usually treated in public hospitals may have been moved to private for-profit hospitals, where capacities remained mostly unaffected.^19^ COVID-19 lockdowns had a negative association with physical activity intensity levels (vigorous, moderate, walking, and overall), daily sitting time, and the amount of rehabilitation provided to older patients.^20,21^ For these reasons, we might have expected to see an increase in the number of hip fractures after lockdowns had been eased because of a potential higher risk of falls among individuals who had become deconditioned. We did not observe an increase in the incidence of hip fracture after the lockdown period. However, the 81-day postlockdown observation period in our study was limited with regard to assessing changes in hip fracture incidence. Further studies are needed to investigate the long-term consequences of the COVID-19 pandemic on hip fractures.

The decrease in the number of hip fractures was observed across France, even in regional departments with low COVID-19 incidence and mortality rates. Moreover, when analyses were presented by French department, we found no correlation between HRRs for hip fractures and all-cause MRRs. These findings strengthen the hypothesis that the lower number of hip fractures was associated with the stay-at-home strategy and not with excess mortality due to COVID-19.

### Strengths and Limitations

This study has strengths. This population-based study included a large number of patients with hip fracture in the study cohort, which increased its statistical power. Furthermore, we believe that hip fractures (ie, fragility fractures) were properly identified because we used an algorithm for fracture identification.

This study also has limitations. First, the number of other fractures, especially other major osteoporotic fractures, was not analyzed. Second, we did not collect data for all of 2020. Third, we had no data on where (indoors or outdoors) the hip fractures occurred.

## Conclusions

The findings suggest that the first nationwide COVID-19 lockdown in France was associated with a decrease in the absolute number of hip fractures. Further studies are needed to investigate the long-term consequences of the COVID-19 pandemic on the incidence of osteoporotic fractures and the management of osteoporosis.

## Notes

Supplement. **eTable 1.** ICD-10 and CCAM Codes Used in This Study **eTable 2.** Overall Mortality Risk Ratio (MRRs) and Hospitalization Rate Ratio (HRRs) During the Lockdown Period in 2020 by French Departments
eMethods.
**eFigure.** Correlation Between Hospitalization Rate Ratios of Hip Fractures (March 16, 2020 to May 10, 2020 vs March 16, 2019 to May 10, 2019) and All-Cause Mortality Rate Ratios (March 16, 2020 to May 10, 2020 vs March 16, 2019 to May 10, 2019) in French Departments
