# Supplementary material for: Analysis of Hip Fractures in France During the First COVID-19 Lockdown in Spring 2020
Source: JAMA Netw Open. 2021 Nov 17;4(11):e2134972. doi: 10.1001/jamanetworkopen.2021.34972 (PMC8600388; doi:10.1001/jamanetworkopen.2021.34972)
Supplement: Supplement. — eTable 1. ICD-10 and CCAM Codes Used in This Study eTable 2. Overall Mortality Risk Ratio (MRRs) and Hospitalization Rate Ratio (HRRs) During the Lockdown Period in 2020 by French Departments eMethods. eFigure. Correlation Between Hospitalization Rate Ratios of Hip Fractures (March 16, 2020 to May 10, 2020 vs March 16, 2019 to May 10, 2019) and All-Cause Mortality Rate Ratios (March 16, 2020 to May 10, 2020 vs March 16, 2019 to May 10, 2019) in French Departments [file jamanetwopen-e2134972-s001.pdf]

Supplementary Online Content

Paccou J, Lenne X, Ficheur G, Theis D, Cortet B, Bruandet A. Analysis of hip fractures in France during the first COVID-19 lockdown in spring 2020. *JAMA Netw Open*. 2021;4(11):e2134972. doi:10.1001/jamanetworkopen.2021.34972

eTable 1. ICD-10 and CCAM Codes Used in This Study
eTable 2. Overall Mortality Risk Ratio (MRRs) and Hospitalization Rate Ratio (HRRs) During the Lockdown Period in 2020 by French Departments
eMethods.
eFigure. Correlation Between Hospitalization Rate Ratios of Hip Fractures (March 16, 2020 to May 10, 2020 vs. March 16, 2019 to May 10, 2019) and All-Cause Mortality Rate Ratios (March 16, 2020 to May 10, 2020 vs. March 16, 2019 to May 10, 2019) in French Departments

This supplementary material has been provided by the authors to give readers additional information about their work.

© 2021 Paccou J et al. *JAMA Network Open*.

eTable 1. ICD-10 and CCAM codes used in this study

Parameters		ICD-10 or procedure codes
Hip fracture	ICD-10 codes	S7200, S7210, S7220
Hip Surgery	CCAM codes	NEKA010, NEKA011, NEKA012, NEKA013, NEKA014, NEKA015, NEKA016, NEKA017, NEKA018, NEKA019, NEKA020, NEKA021
Bone metastases	ICD-10 codes	C795
Multiple fractures	ICD-10 codes	S0200, S0201, S0210, S0211, S0220, S0221, S0230, S0231, S0240, S0241, S025, S0250, S0251, S0260, S0261, S0270, S0271, S0280, S0281, S0290, S0291, S1200, S1201, S1210, S1211, S1220, S1221, S1270, S1271, S1280, S1281, S1290, S1291, S2200, S2201, S2210, S2211, S2220, S2221, S2230, S2231, S2240, S2241, S2250, S2251, S2280, S2281, S2290, S2291, S3200, S3201, S3210, S3211, S3220, S3221, S3230, S3231, S3240, S3241, S3250, S3251, S3270, S3271, S3280, S3281, S4200, S4201, S4210, S4211, S4220, S4221, S4230, S4231, S4240, S4241, S4270, S4271, S4280, S4281, S4290, S4291, S5200, S5201, S5210, S5211, S5220, S5221, S5230, S5231, S5240, S5241, S5250, S5251, S5260, S5261, S5270, S5271, S5280, S5281, S5290, S5291, S6200, S6201, S6210, S6211, S6220, S6221, S6230, S6231, S6240, S6241, S6250, S6251, S6260, S6261, S6270, S6271, S6280, S6281, S7200, S7201, S7210, S7211, S7220, S7221, S7230, S7231, S7240, S7241, S7270, S7271, S7280, S7281, S7290, S7291, S8200, S8201, S8210, S8211, S8220, S8221, S8230, S8231, S8240, S8241, S8250, S8251, S8260, S8261, S8270, S8271, S8280, S8281, S8290, S8291, S9200, S9201, S9210, S9211, S9220, S9221, S9230, S9231, S9240, S9241, S9250, S9251, S9270, S9271, S9290, S9291, T0200, T0201, T0210, T0211, T0220, T0221, T0230, T0231, T0240, T0241, T0250, T0251, T0260, T0270, T0280, T0290, T0291, T08+0, T08+1, T10+0, T12+0, T12+1, T1420, T1421, T902, T911, T912, T921, T922, T931, T932
Diabetes comorbidity	ICD-10 codes	E1100, E1108, E1110, E1118, E1120, E1128, E1130, E1138, E1140, E1148, E1150, E1158, E1160, E1168, E1170, E1178, E1180, E1188, E1190, E1198
History of any osteoporotic fracture	ICD-10 codes	M800, M8000, M8001, M8002, M8003, M8004, M8005, M8006, M8007, M8008, M8009, M801, M8010, M8011, M8012, M8013, M8014, M8015, M8016, M8017, M8018, M8019, M802, M8020, M8021, M8022, M8023, M8024, M8025, M8026, M8027, M8028, M8029, M803, M8030, M8031, M8032, M8033, M8034, M8035, M8036, M8037, M8038, M8039, M804, M8040, M8041, M8042, M8043, M8044, M8045, M8046, M8047, M8048, M8049, M805, M8050, M8051, M8052, M8053, M8054, M8055, M8056, M8057, M8058, M8059, M808, M8080, M8081, M8082, M8083, M8084, M8085, M8086, M8087, M8088, M8089, M809, M8090, M8091, M8092, M8093, M8094, M8095, M8096, M8097, M8098, M8099, S2200, S2240, S3200, S3210, S3220, S3230, S3240, S3250, S3270, S3280, S4220, S7200, S7210, S7220
COPD comorbidity	ICD-10 codes	J440, J441, J448, J449
CVD comorbidity	ICD-10 codes	G450, G451, G452, G453, G454, G458, G459, I200, I200+0, I201, I208, I209, I2100, I21000, I2108, I2110, I21100, I2118, I2120, I21200, I2128, I2130, I21300, I2138, I2140, I21400, I2148, I2190, I21900, I2198, I2200, I22000, I2208, I2210, I22100, I2218, I2280, I22800, I2288, I2290, I22900, I2298, I252, I600, I601, I602, I603, I604, I605, I606, I607, I608, I609, I610, I611, I612, I613, I614, I615, I616, I618, I619, I620, I621, I629, I630, I631, I632, I633, I634, I635, I636, I638, I639, I64, I690, I691, I692, I693, I694, I698

COPD: chronic obstructive pulmonary disease
CVD: cardiovascular disease

© 2021 Paccou J et al. *JAMA Network Open*.

eTable 2. Overall mortality risk ratio (MRRs) and hospitalization rate ratio (HRRs) during the lockdown period in 2020 by French departments.

	Overall mortality					Hospitalization				
	2019	2020	MRRs	95% CI	p	2019	2020	HRRs	95% CI	p
Ain	677	833	1.23	[1.11;1.36]	<0.001	63	55	0.87	[0.61 ; 1.25]	0.462
Aisne	823	1,263	1.53	[1.41;1.68]	<0.001	79	70	0.89	[0.64 ; 1.22]	0.461
Allier	694	712	1.03	[0.92;1.14]	0.631	99	82	0.83	[0.62 ; 1.11]	0.207
Alpes-de-Haute-Provence	255	272	1.07	[0.90;1.27]	0.459	42	39	0.93	[0.60 ; 1.44]	0.739
Hautes-Alpes	209	216	1.03	[0.85;1.25]	0.734	51	35	0.69	[0.45 ; 1.06]	0.086
Alpes-Maritimes	1,794	1,981	1.10	[1.04;1.18]	0.002	298	280	0.94	[0.80 ; 1.11]	0.454
Ardèche	534	711	1.33	[1.19;1.49]	<0.001	67	84	1.25	[0.91 ; 1.73]	0.167
Ardennes	417	468	1.12	[0.98;1.28]	0.087	36	49	1.36	[0.89 ; 2.09]	0.160
Ariège	273	262	0.96	[0.81;1.14]	0.634	40	45	1.13	[0.73 ; 1.72]	0.588
Aube	493	577	1.17	[1.04;1.32]	0.010	48	50	1.04	[0.70 ; 1.55]	0.840
Aude	608	695	1.14	[1.03;1.27]	0.016	111	63	0.57	[0.42 ; 0.77]	<0.001
Aveyron	513	552	1.08	[0.95;1.21]	0.232	84	75	0.89	[0.65 ; 1.22]	0.476
Bouches-du-Rhône	2,838	3,490	1.23	[1.17;1.29]	<0.001	438	361	0.82	[0.72 ; 0.95]	0.007
Calvados	1,184	1,164	0.98	[0.91;1.07]	0.680	116	99	0.85	[0.65 ; 1.12]	0.247
Cantal	307	314	1.02	[0.87;1.20]	0.779	48	41	0.85	[0.56 ; 1.30]	0.459
Charente	671	659	0.98	[0.88;1.10]	0.742	82	74	0.90	[0.66 ; 1.24]	0.522
Charente-Maritime	1,142	1,188	1.04	[0.96;1.13]	0.341	157	134	0.85	[0.68 ; 1.07]	0.178
Cher	552	640	1.16	[1.03;1.30]	0.011	33	50	1.52	[0.98 ; 2.35]	0.064
Corrèze	488	515	1.06	[0.93;1.19]	0.394	70	78	1.11	[0.81 ; 1.54]	0.511
Côte-d'Or	864	1,125	1.30	[1.19;1.42]	<0.001	107	91	0.85	[0.64 ; 1.12]	0.256
Côtes-d'Armor	1,114	1,118	1.00	[0.92;1.09]	0.933	124	103	0.83	[0.64 ; 1.08]	0.164
Creuse	294	286	0.98	[0.83;1.15]	0.740	20	19	0.95	[0.51 ; 1.78]	0.873
Dordogne	753	714	0.95	[0.86;1.05]	0.309	103	100	0.97	[0.74 ; 1.28]	0.833
Doubs	625	1,050	1.68	[1.52;1.85]	<0.001	86	75	0.87	[0.64 ; 1.19]	0.386
Drôme	727	900	1.24	[1.12;1.37]	<0.001	111	92	0.83	[0.63 ; 1.09]	0.183
Eure	665	869	1.31	[1.18;1.45]	<0.001	49	59	1.20	[0.82 ; 1.76]	0.337
Eure-et-Loir	587	808	1.38	[1.24;1.53]	<0.001	55	65	1.18	[0.83 ; 1.69]	0.362
Finistère	1,543	1,637	1.06	[0.99;1.14]	0.096	191	182	0.95	[0.78 ; 1.17]	0.641
Corse-du-Sud	203	249	1.23	[1.02;1.48]	0.031	32	23	0.72	[0.42 ; 1.23]	0.227
Haute-Corse	254	242	0.95	[0.80;1.14]	0.590	41	32	0.78	[0.49 ; 1.24]	0.293
Gard	1,040	1,160	1.12	[1.03;1.21]	0.011	160	159	0.99	[0.80 ; 1.24]	0.955
Haute-Garonne	1,615	1,629	1.01	[0.94;1.08]	0.806	256	237	0.93	[0.78 ; 1.10]	0.392
Gers	335	381	1.14	[0.98;1.32]	0.086	46	36	0.78	[0.51 ; 1.21]	0.271
Gironde	2,228	2,268	1.02	[0.96;1.08]	0.551	309	289	0.94	[0.80 ; 1.10]	0.414
Hérault	1,720	1,865	1.08	[1.02;1.16]	0.015	259	225	0.87	[0.73 ; 1.04]	0.123
Ille-et-Vilaine	1,355	1,434	1.06	[0.98;1.14]	0.135	167	175	1.05	[0.85 ; 1.30]	0.665
Indre	440	625	1.42	[1.26;1.60]	<0.001	65	46	0.71	[0.49 ; 1.03]	0.073
Indre-et-Loire	905	976	1.08	[0.99;1.18]	0.102	101	93	0.92	[0.69 ; 1.22]	0.566
Isère	1,409	1,582	1.12	[1.04;1.21]	0.002	191	174	0.91	[0.74 ; 1.12]	0.374
Jura	368	483	1.32	[1.15;1.51]	<0.001	52	49	0.94	[0.64 ; 1.39]	0.765
Landes	586	602	1.03	[0.92;1.15]	0.643	79	84	1.06	[0.78 ; 1.45]	0.695
Loir-et-Cher	584	662	1.13	[1.01;1.27]	0.027	77	64	0.83	[0.60 ; 1.16]	0.274
Loire	1,303	1,573	1.21	[1.12;1.30]	<0.001	168	152	0.91	[0.73 ; 1.13]	0.371
Haute-Loire	374	334	0.89	[0.77;1.04]	0.133	43	40	0.93	[0.60 ; 1.43]	0.742
Loire-Atlantique	1,919	2,055	1.07	[1.01;1.14]	0.031	247	215	0.87	[0.73 ; 1.05]	0.137
Loiret	912	1,040	1.14	[1.04;1.25]	0.004	95	104	1.10	[0.83 ; 1.45]	0.524
Lot	318	378	1.19	[1.02;1.38]	0.023	47	45	0.96	[0.64 ; 1.44]	0.835
Lot-et-Garonne	568	643	1.13	[1.01;1.27]	0.031	87	82	0.94	[0.70 ; 1.27]	0.701
Lozère	129	140	1.09	[0.85;1.38]	0.503	20	14	0.70	[0.35 ; 1.39]	0.306
Maine-et-Loire	1,111	1,258	1.13	[1.04;1.23]	0.003	140	121	0.86	[0.68 ; 1.10]	0.240
Manche	842	941	1.12	[1.02;1.23]	0.019	94	105	1.12	[0.85 ; 1.48]	0.436
Marne	853	1240	1.45	[1.33;1.59]	<0.001	80	83	1.04	[0.76 ; 1.41]	0.814
Haute-Marne	300	426	1.42	[1.22;1.65]	<0.001	53	35	0.66	[0.43 ; 1.01]	0.057
Mayenne	438	476	1.09	[0.95;1.24]	0.209	56	56	1.00	[0.69 ; 1.45]	1.000
Meurthe-et-Moselle	1,092	1,493	1.37	[1.27;1.48]	<0.001	118	103	0.87	[0.67 ; 1.14]	0.313
Meuse	343	469	1.37	[1.19;1.57]	<0.001	36	26	0.72	[0.44 ; 1.20]	0.206
Morbihan	1,297	1,376	1.06	[0.98;1.14]	0.127	205	172	0.84	[0.69 ; 1.03]	0.090
Moselle	1,572	2,555	1.63	[1.53;1.73]	<0.001	191	145	0.76	[0.61 ; 0.94]	0.012
Nièvre	418	475	1.14	[1.00;1.30]	0.057	47	42	0.89	[0.59 ; 1.35]	0.596
Nord	3,492	4,264	1.22	[1.17;1.28]	<0.001	387	319	0.82	[0.71 ; 0.96]	0.011
Oise	923	1,512	1.64	[1.51;1.78]	<0.001	101	99	0.98	[0.74 ; 1.29]	0.888
Orne	500	530	1.06	[0.94;1.20]	0.350	68	67	0.99	[0.70 ; 1.38]	0.931
Pas-de-Calais	2,120	2,491	1.18	[1.11;1.25]	<0.001	240	203	0.85	[0.70 ; 1.02]	0.079
Puy-de-Dôme	1,008	1,048	1.04	[0.95;1.13]	0.378	109	91	0.84	[0.63 ; 1.10]	0.204

© 2021 Paccou J et al. *JAMA Network Open*.

Pyrénées-Atlantiques	1,148	1,169	1.02	[0.94;1.10]	0.663	201	166	0.83	[0.67 ; 1.01]	0.068
Hautes-Pyrénées	403	484	1.20	[1.05;1.37]	0.007	71	60	0.85	[0.60 ; 1.19]	0.337
Pyrénées-Orientales	917	938	1.02	[0.93;1.12]	0.626	147	96	0.65	[0.50 ; 0.84]	0.001
Bas-Rhin	1,504	2,450	1.63	[1.53;1.74]	<0.001	206	180	0.87	[0.72 ; 1.07]	0.186
Haut-Rhin	1,071	2,345	2.19	[2.04;2.35]	<0.001	109	135	1.24	[0.96 ; 1.59]	0.097
Rhône	2,259	3,232	1.43	[1.36;1.51]	<0.001	297	261	0.88	[0.74 ; 1.04]	0.128
Haute-Saône	334	452	1.35	[1.17;1.56]	<0.001	33	34	1.03	[0.64 ; 1.66]	0.903
Saône-et-Loire	1,034	1,172	1.13	[1.04;1.23]	0.003	129	117	0.91	[0.71 ; 1.16]	0.444
Sarthe	837	973	1.16	[1.06;1.28]	0.001	82	85	1.04	[0.77 ; 1.40]	0.816
Savoie	563	673	1.20	[1.07;1.34]	0.002	140	75	0.54	[0.40 ; 0.71]	<0.001
Haute-Savoie	867	1,169	1.35	[1.23;1.47]	<0.001	166	119	0.72	[0.57 ; 0.91]	0.006
Paris	2,603	4,693	1.80	[1.72;1.89]	<0.001	478	359	0.75	[0.65 ; 0.86]	<0.001
Seine-Maritime	1,936	2,264	1.17	[1.10;1.24]	<0.001	214	175	0.82	[0.67 ; 1.00]	0.048
Seine-et-Marne	1,190	2,299	1.93	[1.80;2.07]	<0.001	165	141	0.86	[0.68 ; 1.07]	0.171
Yvelines	1,291	2,295	1.78	[1.66;1.90]	<0.001	167	158	0.95	[0.76 ; 1.18]	0.618
Deux-Sèvres	539	623	1.16	[1.03;1.30]	0.014	63	73	1.16	[0.83 ; 1.62]	0.392
Somme	957	1,242	1.30	[1.19;1.41]	<0.001	82	93	1.13	[0.84 ; 1.53]	0.406
Tarn	652	674	1.03	[0.93;1.15]	0.546	97	101	1.04	[0.79 ; 1.38]	0.776
Tarn-et-Garonne	404	379	0.94	[0.82;1.08]	0.372	57	40	0.70	[0.47 ; 1.05]	0.086
Var	1,635	1,843	1.13	[1.05;1.20]	<0.001	241	229	0.95	[0.79 ; 1.14]	0.580
Vaucluse	940	882	0.94	[0.86;1.03]	0.174	159	130	0.82	[0.65 ; 1.03]	0.089
Vendée	1,067	1,096	1.03	[0.94;1.12]	0.533	159	146	0.92	[0.73 ; 1.15]	0.457
Vienne	740	758	1.02	[0.93;1.13]	0.642	89	84	0.94	[0.70 ; 1.27]	0.704
Haute-Vienne	748	689	0.92	[0.83;1.02]	0.120	80	68	0.85	[0.62 ; 1.17]	0.324
Vosges	561	1,053	1.88	[1.69;2.08]	<0.001	73	69	0.95	[0.68 ; 1.31]	0.737
Yonne	635	754	1.19	[1.07;1.32]	0.001	79	77	0.98	[0.71 ; 1.33]	0.873
Territoire de Belfort	353	541	1.53	[1.34;1.75]	<0.001	64	61	0.95	[0.67 ; 1.35]	0.788
Essonne	1,071	2,298	2.15	[2.00;2.31]	<0.001	147	134	0.91	[0.72 ; 1.15]	0.438
Hauts-de-Seine	1,504	3,434	2.28	[2.15;2.43]	<0.001	119	99	0.83	[0.64 ; 1.09]	0.176
Seine-Saint-Denis	1,132	2,764	2.44	[2.28;2.62]	<0.001	111	106	0.96	[0.73 ; 1.25]	0.734
Val-de-Marne	1,475	3,065	2.08	[1.95;2.21]	<0.001	105	88	0.84	[0.63 ; 1.11]	0.222
Val-d'Oise	1,127	2,394	2.12	[1.98;2.28]	<0.001	169	104	0.62	[0.48 ; 0.79]	<0.001

MRR: mortality rate ratios

HRR: hospitalization rate ratios

© 2021 Paccou J et al. *JAMA Network Open*.

eMethods

For each parameter and each period, Poisson regression fits models of the number of hip fractures. A dummy-year variable equal to 0 or 1, for cases in 2019 and 2020, respectively, was introduced in the model as an independent variable. Considering the three periods, the overall case and the eleven parameters – gender (male, female), age group (50–59, 60–69, 70-79, 80-89, over 89 years) and hospital type (public university hospitals, public hospitals, private for-profit hospitals and private non-profit hospitals –, thirty six estimations was performed.

For example, for the overall population:

The Stata estimation commands for the lockdown period were:

```
poisson nb_fractures yr2020 if period == "Lockdown ", irr
```

We used the "irr" option to obtain the incidence-rate ratios rather than the underlying coefficients.

The Stata output was:

```
Poisson regression                              Number of obs   =          2
                                                LR chi2(1)      =      82.47
                                                Prob > chi2     =     0.0000
Log likelihood = -11.151229                     Pseudo R2       =     0.7871

------------------------------------------------------------------------------
 nb_fractures|        IRR   Std. Err.      z    P>|z|     [95% Conf. Interval]
-------------+----------------------------------------------------------------
      yr2020 |   .8851638   .0119008    -9.07   0.000     .8621433     .908799
       _cons |      11782   108.5449  1017.54   0.000     11571.17    11996.68
------------------------------------------------------------------------------
```

The data are:

period	yr2020	nb_fractures
Pre-lockdown	0	17,023
Pre-lockdown	1	16,729
Lockdown	0	11,782
Lockdown	1	10,429
Post-lockdown	0	17,588
Post-lockdown	1	17,609

© 2021 Paccou J et al. *JAMA Network Open*.

eFigure. Correlation between hospitalization rate ratios of hip fractures (March 16, 2020 to May 10, 2020 vs. March 16, 2019 to May 10, 2019) and all-cause mortality rate ratios (March 16, 2020 to May 10, 2020 vs. March 16, 2019 to May 10, 2019) in French departments. Each dot represents a French department.

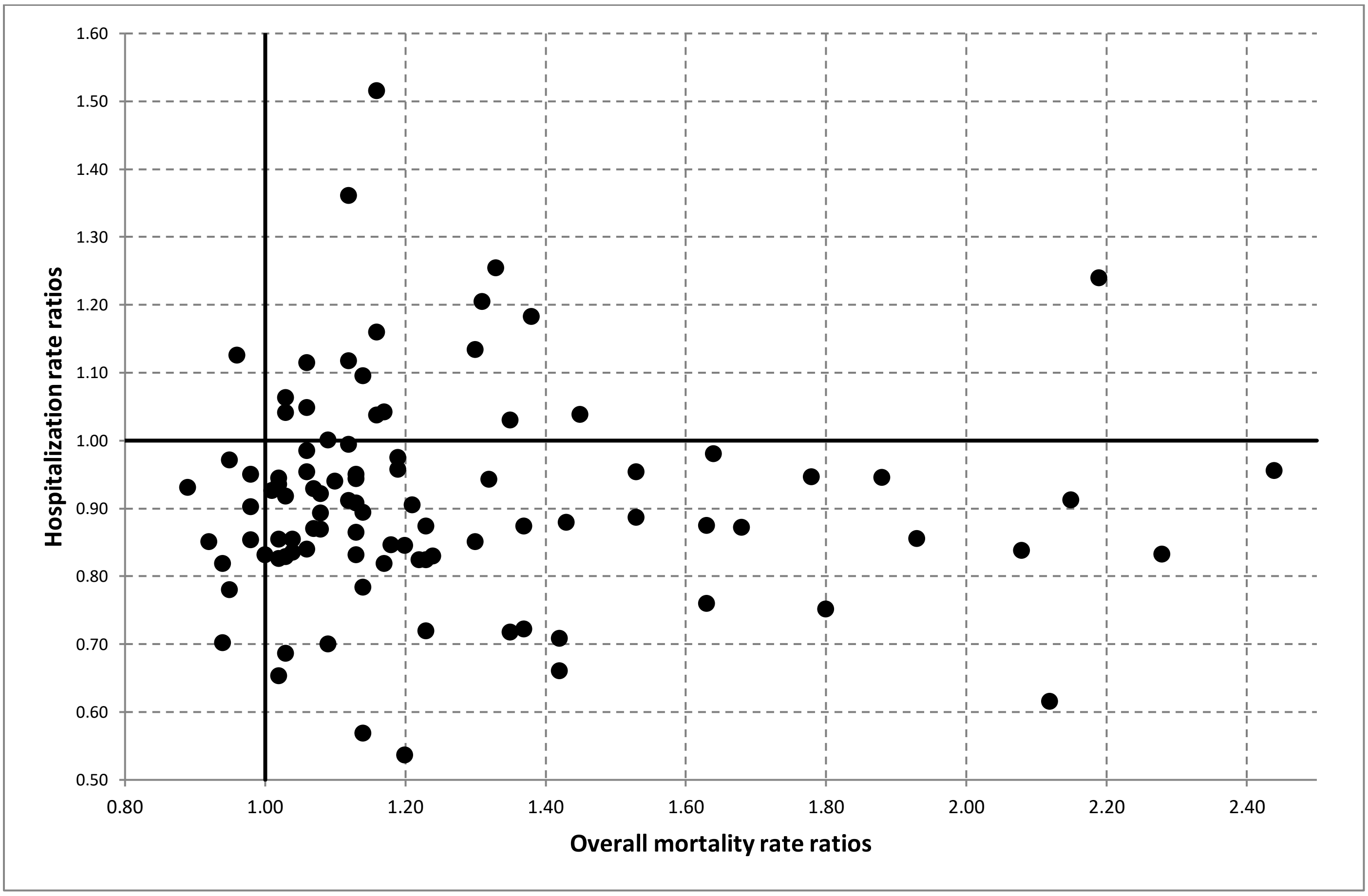

© 2021 Paccou J et al. *JAMA Network Open*.
